# The Roles of Optogenetics and Technology in Neurobiology: A Review

**DOI:** 10.3389/fnagi.2022.867863

**Published:** 2022-04-19

**Authors:** Wenqing Chen, Chen Li, Wanmin Liang, Yunqi Li, Zhuoheng Zou, Yunxuan Xie, Yangzeng Liao, Lin Yu, Qianyi Lin, Meiying Huang, Zesong Li, Xiao Zhu

**Affiliations:** ^1^Department of Laboratory Medicine, Hangzhou Medical College, Hangzhou, China; ^2^Zhu’s Team, Guangdong Medical University, Zhanjiang, China; ^3^Department of Biology, Chemistry, Pharmacy, Free University of Berlin, Berlin, Germany; ^4^Guangdong Provincial Key Laboratory of Systems Biology and Synthetic Biology for Urogenital Tumors, Shenzhen Key Laboratory of Genitourinary Tumor, Department of Urology, The First Affiliated Hospital of Shenzhen University, Shenzhen Second People’s Hospital (Shenzhen Institute of Translational Medicine), Shenzhen, China

**Keywords:** nanoparticles, nervous system, neural circuits, neurobiology, neuron, optogenetics

## Abstract

Optogenetic is a technique that combines optics and genetics to control specific neurons. This technique usually uses adenoviruses that encode photosensitive protein. The adenovirus may concentrate in a specific neural region. By shining light on the target nerve region, the photosensitive protein encoded by the adenovirus is controlled. Photosensitive proteins controlled by light can selectively allow ions inside and outside the cell membrane to pass through, resulting in inhibition or activation effects. Due to the high precision and minimally invasive, optogenetics has achieved good results in many fields, especially in the field of neuron functions and neural circuits. Significant advances have also been made in the study of many clinical diseases. This review focuses on the research of optogenetics in the field of neurobiology. These include how to use optogenetics to control nerve cells, study neural circuits, and treat diseases by changing the state of neurons. We hoped that this review will give a comprehensive understanding of the progress of optogenetics in the field of neurobiology.

## Introduction

**FIGURE 1:**
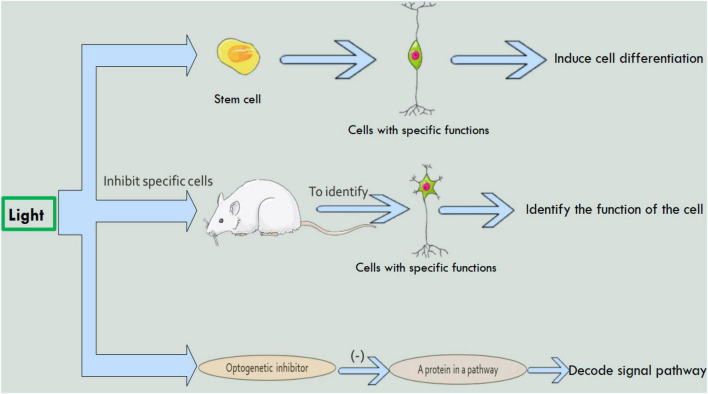
The study of optogenetics in the field of neurons. Using optogenetics, it is possible to induce stem cell differentiation, identify cell function, and decode intercellular signaling pathways.

**FIGURE 2:**
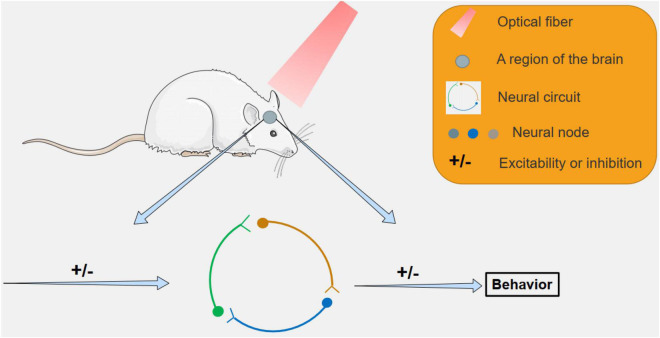
The study of neural circuits by optogenetic methods. By targeting specific areas with light, specific neural circuits can be inhibited or activated, leading to behavioral changes in mice, and related neural circuits can be studied.

**FIGURE 3:**
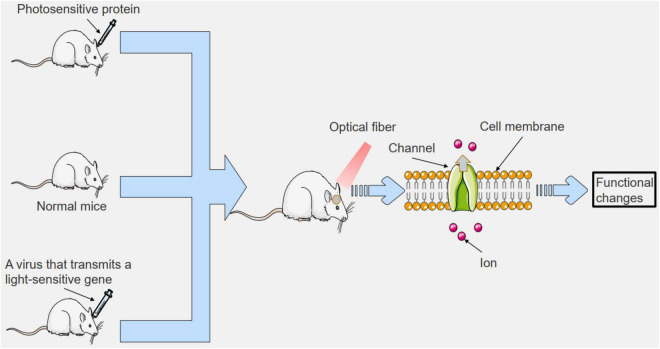
To study the mechanisms of clinical diseases using optogenetics. We can introduce photosensitive proteins outside the body, or we can introduce viruses that transmit photosensitive genes, or we can shine light directly on specific areas. This can lead to changes in cell membrane pathways in the irradiated area, which can affect cell function. The related functions of organisms can be altered to study clinical diseases.

In 2005, optogenetics was born and appeared in the public (Boyden et al., 2005). Since the advent of optogenetics, many top medical journals have described it as a core technology for the future of humanity (Method of the year, 2010; News, 2010; Adamantidis et al., 2015). Optogenetics can be combined with molecular biology, viral biology and other methods to introduce foreign light-sensitive protein genes into living cells (Amitrano et al., 2021; Di Ventura and Weber, 2021). Therefore, optogenetics has made many achievements in the field of neurobiology. Such as exploring unknown neuron functions (Figure 1), the discovery of neural circuits (Figure 2), and treatment of neurological diseases (Figure 3). By using the technology of optogenetics, the research of many difficult diseases has been advanced greatly. Using optogenetics to induce the differentiation of neural progenitor cells, the researchers were able to treat stroke in mice (Yu et al., 2019; Peters et al., 2021; Vassalli et al., 2021). The mice were able to remove and reactivate a memory by changing the connections between related neurons in the brain with different frequencies of light (Nabavi et al., 2014). Blindness can be treated by using optogenetics to activate the photogene expression of the related photoactivation channels or pumps in retinal cells (Ostrovsky and Kirpichnikov, 2019). This technology have led us into a higher research field.

## Optogenetic Research Methods

### Nerve Cells

#### Induce Neuronal Differentiation

In 2019, Luo et al. (2019) used photoelectric fullerene-bound photosensitive protein (HEBR) to reprogram and differentiate human fibroblasts. In this study, researchers transfected HEBR plastids into human fibroblasts using fullerene as a cell culture substrate (Figure 1). Previous research has shown that environmental stresses, such as acidity, can stimulate cell reprogramming. When the researchers illuminated HERB transfected fibroblasts with green light, the pH in and out of the transfected fibroblasts changed momentously, causing the fibroblasts to differentiate into neuron-like cells. This study has clinical significance in nerve repair.

In other studies, researchers combined a variety of technologies, such as optogenetics, synthetic biology (Padmanabhan et al., 2019; Hemmati et al., 2020; Yi et al., 2020; Zhang et al., 2020), for the first time to achieve far red light to control the expression of genomic genes. The far-red light-regulated CRISPR-dCas9 endogenous gene transcription activation device was developed for the first time (Gjaltema and Schulz, 2018), which successfully induced the pluripotent stem cells into functional neural cells (Shao et al., 2018). By combining BphS, which respond to red light proteins in rhodobacter, transcription factors BldD in streptococcus, and protein dCas9 in streptococcus pyogenes, this device can accurately realize the reversible activation of target genes inside and outside the organism, with high precision. Meanwhile, the frequency of light used in the study is in the physiological range and has no side effects on organisms. In theory, the results of this research can be widely used for precise epigenetic regulation (Hori and Kikuchi, 2019). And, in the future, this technology may be applied in the clinical field to treat diseases such as muscular dystrophy (Vajtay et al., 2019).

#### Control the Behavior of Nerve Cells

In earlier studies, optogenetics simply stimulated neurons (Nowak et al., 2010). Early applications of optogenetic control neurons were mainly two: driving proton pumps with light to charge mitochondria (Hallett et al., 2016), and polarizing or depolarizing neurons (Yao et al., 2012). By making a single neuron hyperpolarized, the function of this neuron can be studied (Aquili et al., 2014). In the latest study, researchers can decode and control signaling pathways in neurons (Figure 1; Melero-Fernandez de Mera et al., 2017). In this study, the intracellular signaling pathways of the organism can be controlled by light, using the main bioresonance effect of the organism. Using light to control specific signaling pathways that regulate the behavior of nerve cells, researchers can learn which neurons are involved in those pathways. In related studies, we can learn more about the JNK signaling pathway by inhibiting p38MAPK with OptoJNKI (a photosensitive substance that inhibits p38MAPK) (Melero-Fernandez de Mera et al., 2017). Moreira et al. (2019) managed to control the taste of fruit flies by shining different LEDs on different taste neurons. In this study, the researchers managed to alter feeding behavior in fruit flies by manipulating taste receptors. This technique can be used to study the progression of clinical diseases and to discover new therapies.

#### Study Neuronal Function

Studies have shown that people with schizophrenia and other psychiatric and neurological disorders have gamma oscillations in their brains (Gao et al., 2021a,b). But exactly how gamma oscillations are produced is not clear (Fan et al., 2020; Lu W. et al., 2020; Song et al., 2020). Cardin et al. (2009) discovered how the brain produces gamma oscillations by using optogenetics to manipulate the activity of nerve cells. By manipulating the interneurons’ related behavior with different frequencies of light, the researchers were able to observe the extent of the gamma oscillations produced by the interneurons. The research will contribute to a range of neurological disorders.

In 2014, researchers used optogenetics to inactivate cells in parts of the rat brain (Figure 1) to identify the neurons responsible for behavioral decision-making (Aquili et al., 2014). This study is the first to show that optogenetics inhibition of nucleus accumbens neurons during reward and false feedback can increase the behavioral complexity of individuals (Aquili et al., 2014). In the same year, another researcher used optogenetics to identify neurons that control aggression in the hypothalamus of mice (Lee et al., 2014). In 2017, scientists used optogenetics to find neurons in the brains of mice that control hunting behavior (Han et al., 2017). Because the hypothalamus in humans and mice is structurally similar, these findings are also useful for studying human behavior.

### Neural Circuits

#### The Neural Circuits That Regulate Sodium Appetite

Sodium ions are important ions in the nervous system that regulate neurons. If sodium ion is not ingested for a long time, it will cause symptoms such as loss of appetite, weakness of limbs and dizziness. When a variety of animals are deficient in sodium ions, they will consume a large amount of salt rich in sodium ions, which is called sodium appetite (Thornton and Fitzsimons, 1995; Geerling and Loewy, 2008; Molnar and Labouesse, 2021). After a large intake of salt, the body will produce a sense of satisfaction to prevent further intake (Wolf et al., 1984). Previous studies on sodium appetite were flawed and did not conform to the single variable principle of the experiment. Until 2019, Lee et al. (2019) demonstrated that the pre-LCPDYN neurons are the core neurons in the regulation of sodium appetite by combining optogenetics with other techniques, and are regulated by the homeostatic and sense-related brain regions (Table 1 and Figure 2). In this study, researchers used optogenetic techniques to inhibit pre-LCPDYN neurons, and found that the pre-LCPDYN neurons are essential in the neural circuits that regulate sodium appetite. This experiment has revealed the mechanism of nerve circuits regulating sodium appetite and related conclusions.

**TABLE 1:** The study of optogenetics in neurobiology.

The field of optogenetics	The specific research		References
Nerve cells	Induce neuronal differentiation		Shao et al., 2018
	Control the behavior of nerve cells		Moreira et al., 2019
	Study neuronal function		Aquili et al., 2014
Neural circuits	The neural circuits that regulate sodium appetite		Lee et al., 2019
	The nerve basis of compulsive feeding		Nieh et al., 2015; Kim et al., 2021
	Social behavioral neural circuits		Zhao et al., 2017; Yang Y. et al., 2021
	Body temperature regulating neural circuit		Zhao et al., 2017
	Spatial learning and memory circuits		Yang et al., 2018; Huang et al., 2021
	Neural circuit mechanisms that activate addictive memory		Alaghband et al., 2014
Nervous system-based clinical research	Nervous system	Alzheimer’s disease	Roy et al., 2016
		Parkinson’s disease	Ztaou et al., 2016
		Epilepsy	Chen et al., 2018
		Stroke	Yu et al., 2019
	Skeletal system		Bryson et al., 2014
	Urinary system		Mickle et al., 2019
	Pain		Samineni et al., 2017; Harriott et al., 2021
	Vision		Berry et al., 2019; Gilhooley et al., 2021
	Memory		Vetere et al., 2019

#### The Nerve Basis of Compulsive Feeding

Globally, obesity (Hosseinpanah et al., 2019; Lu D. et al., 2020) and type 2 diabetes (Broder et al., 2014; Song et al., 2020; Liu Y. et al., 2021) are among the major diseases that endanger human health. Bad eating habits can bring about many diseases. Clinically, the treatment for severely obese patients is usually gastric bypass surgery. This approach is extremely traumatic. Optogenetics separates normal eating behavior from reward-seeking eating (Cardi et al., 2018), providing new ideas for the treatment of this disease.

Nieh et al. (2015) demonstrated that the hypothalamic-ventral tegmental pathway is involved in controlling feeding in starving mice by activating or inhibiting specific neurons using optogenetics (Jennings et al., 2015; Table 1 and Figure 2). In this study, the researchers introduced light-sensitive proteins that control the activity of neurons into the lateral hypothalami-ventral tegmental region (VTA) and activated the region with light, causing already satiated mice to take longer to eat.

In another study, Sternson et al. successfully distinguished appetitive behavior (Sternson and Atasoy, 2014) from neurons that satisfy behavior (Atasoy et al., 2012; Gatto and Goulding, 2018). In the study, the mice were given food freely or a reward for completing a task. Neuronal activity in the lateral hypothalamus of mice was also imaged. Based on this study, researchers were able to identify the neural basis of compulsive eating.

In 2021, the researchers used an AVV virus vector to deliver the ChR2 light-sensitive protein gene to a specific vagus nerve in the stomach (Kim et al., 2021; Table 1 and Figure 2). By using a tiny LED was inserted into the end of a flexible shaft in the stomach. Mice were successfully induced to feel full by external stimulation of specific gastric vagus nerve with remote radio frequency source. In 2022, researchers used optogenetics to inhibit neuropod cells in mice intestinal mucosa and found that mice consumed less sucrose (Buchanan et al., 2022). These studies suggest that optogenetics has a role to play in understanding the neural circuit of compulsive feeding.

#### Social Behavioral Neural Circuits

Social behavior is one of the characteristics of biology, but the social behavior neural circuits of biology is hardly understood. But through optogenetics, scientists are unraveling the mysteries of biological social behavior. Chiang and his colleagues developed an automatic laser tracking and optogenetic manipulation system (Hsiao et al., 1408; Wu et al., 2014) (ALTOMS) that can be used to study the social memory of fruit flies. After the expression of photosensitive proteins in neurons at specific sites, neurons involved in pain expression photosensitive pathways can be activated when laser irradiation is applied to specific sites (Karim et al., 2006; Table 1 and Figure 2). Using the system, the researchers were able to get certain males to quickly learn to avoid females, while other males continued to approach. At the same time, automated laser tracking and optogenetic manipulation systems (ALTOMS) (Hsiao et al., 1408; Wu et al., 2014) are expected to help identify the neural circuits responsible for specific drosophila behavior and understand the circuitry behind the ability to form memories based on social interaction learning.

In the latest study, researchers implanted tiny wireless optogenetic electronic devices into the brains of mice. The synchrony between brain neurons in the medial prefrontal cortex of mice induced social preference in mice (Yang Y. et al., 2021; Table 1 and Figure 2). The micro technique used in this study was less invasive and had less effect on the natural behavior of mice. Now, optogenetics not only controls the behavior of rodents but also primates (Rajalingham et al., 2021). These studies demonstrates the broad application of optogenetics in human social behavior circuits.

#### Body Temperature Regulating Neural Circuits

Thermoregulation is important for many life activities (Davison, 1976). Human beings have known for the last century that the thermoregulatory center is located in the hypothalamus (Hamilton and Ciaccia, 1971), but it is difficult to use traditional methods to analyze the thermoregulatory mechanism. In order to elucidate the neurons and neural circuits of hypothalamus involved in body temperature regulation, relevant researchers used optogenetics combined with physiological calcium signal recording (Tretyn, 1999) and other means to conduct experiments from the level of neural circuits on the hypothalamus of mice (Zhao et al., 2017). This study found neurons in the preoptic region of the hypothalamus that regulate thermally driven cooling behavior, as well as neurons in the dorsolateral part of the dorsolateral part of the hypothalamus responsible for the thermogenesis mechanism caused by cold stimulation. By using optogenetics to activate vLPO neurons, the researchers found that vLPO neurons are at the core neuron in thermoregulatory neural circuit (Table 1 and Figure 2; Zhao et al., 2017). In addition, a new marker for heat-sensitive neurons, brain-derived neurotrophic factor (BDNF), was identified (Ciszowski et al., 2016). This study provides new clues for physiological and pathological research based on thermoregulation.

#### Spatial Learning and Memory Circuits

Using techniques such as optogenetics, single synapse tracing (Spreafico et al., 1981) and *in vivo* multi-channel electrophysiological recording (Brozoski et al., 2006), the researchers found that the excitatory pyramidal cells of the entorhinal cortex (ECIIPN) (Shu et al., 2016) formed a single synaptic connection(eciipn-ca1pv synapses) with the inhibitory small abruption protein cells of the hippocampus CA1 region (CA1PV) (Shu et al., 2016; Yang et al., 2018). In the transgenic mouse model of Alzheimer’s disease, this memory loop is selectively damaged (Yang et al., 2018). Researchers used optogenetics stimulation therapy to repair eciipn-ca1pv synaptic degeneration damage and effectively treat memory loss in Alzheimer’s disease (Table 1 and Figure 2). This experiment proves that this loop is involved in regulating spatial learning and memory (Yang et al., 2018). In another study, researchers combined optogenetic technology with multi-channel synchronous optical stimulation and electrical recording technology, and found a circuit of emotional influence on spatial learning and memory in terms of structure and function (Zhang et al., 2017). Vahaba et al. (2017) used optogenetics to manipulate the NIF and HVC regions in the brains of zebra finches (Spierings and ten Cate, 2014). By controlling the interaction of these two regions, the researchers managed to encode the finches’ memories (Vahaba et al., 2017). Huang et al. (2021) (Table 1 and Figure 2), Huang et al. treated mice with phototherapy and recorded the potential changes in the hippocampal CA1 region of mice. The results showed that light therapy improved spatial memory and was associated with changes in the activity patterns of hippocampal neurons. These studies on spatial learning and memory could provide insights into the treatment of Alzheimer’s disease and some psychiatric disorders.

#### Neural Circuit Mechanisms That Activate Addictive Memory

Drug addiction (Martinez-Gonzalez et al., 2016; Xu et al., 2021) is an abnormal learning and memory process. Withdrawal scenarios can reactivate the addictive memory when the patient enters a scene that was previously associated with withdrawal symptoms (Hellemans et al., 2006). According to previous studies, the basolateral amygdala (BLA) plays an important role in inducing addictive memory retrieval (Wang et al., 2014; Khakpoor et al., 2016; Yi et al., 2021). However, its downstream neural circuits remain unknown. In this study, researchers combined neural tracer, optogenetics, chemical genetics and other methods. It was found that after activation of BLA -PrL loop, PrL was induced to transmit information back to BLA by projecting neurons, so as to activate the increase of Arc protein expression level in another group of BLA neurons and cause the recall of addictive memory (Table 1 and Figure 2; Alaghband et al., 2014). The study revealed the important role of the prefrontal cortex as a hub of the neural circuits in reactivating addictive memories in withdrawal scenarios, providing support for the treatment of drug addiction.

## Nervous System-Based Clinical Research

### Central Nervous System

#### Alzheimer’s Disease

Currently, none of the drugs used to improve cognitive function can fundamentally treat Alzheimer’s disease, but can only alleviate the symptoms. In 2016, Roy’s team successfully restored memory in mice using optogenetics (Table 1 and Figure 3; Roy et al., 2016). In the study, the researchers implanted light-sensitive proteins into the hippocampus of mice with memory loss. In response to light, memory cells in the hippocampus of the mice were activated. The next day, without light, the mice lost their memory again. As shown in the study, the hippocampus of the memory recovery mice established a strong connection with the entorhinal cortex (Meda et al., 2013) which is missing in Alzheimer’s patients (Liu and Zhang, 2019; Vallee et al., 2020). Another study used optogenetics stimulation therapy to repair eciipn-ca1pv synaptic degeneration damage and effectively treat memory damage caused by Alzheimer’s disease (Figure 3; Yang et al., 2018). At the same time, an important research achievement in Alzheimer’s disease is the treatment of cognitive dysfunction with near-infrared bioluminescence (Saltmarche et al., 2017; Arenas et al., 2020). Up to now, many studies have demonstrated that near-infrared bioluminescence therapy can improve cognitive function (Chao, 2019; Kinouchi et al., 2021). The above research may provide ideas for the radical cure of Alzheimer’s disease.

#### Parkinson’s Disease

Parkinson’s disease is a chronic disease with no clinical cure (Guo et al., 2019; Altinoz et al., 2020). However, Parkinson’s disease can be alleviated and treated with optogenetics. Ztaou et al. (2016) found that bilateral activation of indirect pathway MSNs by optogenetics can produce Parkinson’s-like presentation. However, activation of MSNs in the direct pathway alleviates symptoms such as freezing, bradykinesia, and difficulty in initiating movement (Kravitz et al., 2010; de Almeida and da Silva, 2021; Malaquias et al., 2021; Yang X. et al., 2021). Yang et al. (2018) found that the use of optogenetics combined with deep brain stimulation (DBS) (Castano-Candamil et al., 2019) to stimulate the afferent axons of the subthalamic nucleus region at high frequency can significantly treat Parkinson’s disease (Table 1 and Figure 3). Carter et al. (2010) used optogenetic targeting to control the LC-NE region of the cerebral cortex of mice, and were able to treat sleep disorders in mice with Parkinson’s disease (Figure 3). Steinbeck et al. (2015) induced rapid and reversible reactivation of motor defects in mice that had recovered from Parkinson’s motor defects induced by injury (Figure 3). And a recent study showed that photogenetic stimulation of the deep brain can relieve Parkinson’s disease in rats (Ingram et al., 2020; Yu et al., 2020). These studies suggest that optogenetics has great potential in the clinical treatment of Parkinson’s disease.

#### Epilepsy

More than 20% of epileptic patients develop stubborn resistance to epileptic drugs (Zhou et al., 2013; Murawiec et al., 2020), which eventually develops into refractory epilepsy (Martinez-Juarez et al., 2012; Tsai et al., 2021). The researchers injected green light-emitting nanoparticles into the hippocampus of mice (Roet et al., 2019), and irradiated the cranium surface with infrared light, and found that the epileptic neurons of mice were effectively silenced (Table 1 and Figure 3; Chen et al., 2018). The nanoparticles used in this experiment are stable, biocompatible and can be used for a long time. Lu et al. (2016) conducted *in vivo* and *in vitro* experiments, and the excitatory photosensitive protein was expressed in inhibitory neurons to inhibit epileptoid activity up to 70.0 and 82.4%, respectively. These studies indicate that optogenetic techniques are superior to other methods in the treatment of epilepsy.

#### Stroke

Cerebral apoplexy (Ng et al., 2019) is caused by the obstruction of blood flow to the brain tissues caused by vascular obstruction, which often occurs suddenly, with such symptoms as fainting, hemiplegia, and slant of the tongue, with a high mortality and disability rate. There is still a lack of effective treatment drugs, and transplantation of nerve progenitor cells is a good way to restore the function of nerve neurons in the brain (Cabral-Costa and Kowaltowski, 2020; Ostolaza et al., 2020; Roy-O’Reilly et al., 2020). Yu et al. (2019) provided luciferin CTZ to the brain of mice by intranasal administration (Table 1). When CTZ encounters luminescent proteins, it emits the required light. This study showed that survival rates for the growth and differentiation of neural progenitor cells increased significantly, more intact axons and nerve connections were produced, and better responses to electrical stimulation were achieved. The affected limb also showed better recovery. In young mice, stroke affected limb function was restored to normal levels, and even in older mice, stroke symptoms were partially recovered (Table 1 and Figure 3). The findings offer hope for an effective treatment for stroke.

#### Memory

A memory can bring either pleasure or fear (Kirmayer et al., 1995; Riksen and Netea, 2020). And relevant neurobiological studies have shown that an experience can cause changes in multiple brain areas, such as the cerebral cortex, hippocampus and amygdala, thus producing memory (Josselyn et al., 2015). In the following studies, optogenetics can manipulate memories and erase bad memories by manipulating neurons in the brain. In 2014, researchers successfully removed and reactivated a certain memory by changing the connections of related neurons in the brain of rats with different frequencies of light (Table 1 and Figure 3; Nabavi et al., 2014). In 2017, researchers used optogenetic technology in conjunction with electrophysiological technology and behavioral experiments (Ishikawa and Sakaguchi, 2013) to study the role of specific neural pathways in fear memory (Klavir et al., 2017; Yilmaz et al., 2020). In 2019, relevant studies for the first time found the subgroup of neurons that regulate the new memory of fear extinction, which improved people’s cognition of fear memory (Lacagnina et al., 2019). Also, in 2019, people used optogenetic methods to manipulate memory-related neurons to encode memory imprinting without experience for the first time (Table 1 and Figure 3; Vetere et al., 2019). The above research indicates that optogenetics plays an important role in the study of the mechanism of memory generation and memory-related diseases.

### Peripheral Nervous System

#### Skeletal System

In the past, electric stimulation was often used for patients who lost motor function, and the electric stimulation treatment was prone to muscle fatigue and inaccurate discharge (Asakawa et al., 2021). However, optogenetics can stimulate a certain muscle fiber accurately and with low trauma, which can be used to study the treatment of motor system injury. In 2010, researchers started to apply optogenetics to the treatment of motor impairment (Table 1 and Figure 3). Llewellyn et al. (2010) utilized light stimulation of muscle fibers, and after 20 min of light stimulation of muscle fibers, the muscle still maintained a third of the maximum stress (Table 1 and Figure 3). Bryson et al. (2014) constructed a mouse model of muscle loss innervation and transplanted the embryoid containing ChR2 motor neurons into the mouse. By shining blue light on the transplant site, the researchers were able to restore leg muscle function (Table 1 and Figure 3). Srinivasan et al. (2018) used tiny LED lights to control light-sensitive proteins expressed in the legs of mice. The study succeeded in controlling ankle movement in mice. These studies suggest that optogenetics has great potential in controlling biological motor systems, especially in the treatment of paralysis and the treatment of muscle degeneration.

#### Urinary System

Related researchers developed a full closed-loop optogenetic control system (Mickle et al., 2019) and implanted it into female mice with drug-induced bladder dysfunction. This system can detect bladder filling, and the system can also irradiate the bladder for optogenetic control (Kessler et al., 2019). Shown in the study, photosensitive proteins are expressed in nerve cells of the bladder in mice by optogenetics technique, which makes the neurons in the bladder of mice in a hyperpolarized state. For 7 days after the system was implanted, the mice did well. Finally, the rats returned to normal bladder function (Table 1 and Figure 3; Mickle et al., 2019). Through further research and testing, this method is of clinical value.

#### Pain

Pain is one of the common clinical symptoms. Prolonged severe pain can seriously affect the patient’s quality of life. At present, photogenetic technology can solve the pain problem very well. In 2017, a new in-spinal optogenetics device was used for pain treatment and research (Table 1 and Figure 3; Samineni et al., 2017; Mai et al., 2020). Using this device, the researchers activated the afferent nerve of trpv1-chr2 channel protein, causing pain response behavior in mice (Jacob and Szerb, 1951; Zhu et al., 2020; Ji et al., 2021; Zhang et al., 2021). The researchers then ran a real-time comparison experiment, and the results were the same. Now that the device’s function is clear, it can be widely used in pain research. Another study used the selective silencing of related neurons by a wirelessly controlled electro-optical system to reduce ongoing pain and induced skin allergic reactions in mice under cystitis conditions (Table 1 and Figure 3; Samineni et al., 2017). And it had no bad effect on the mice. Hua et al. (2020) discovered a class of inhibitory neurons called “CeAga.” It turns off pain. Inhibition of the expression of CeAga neurons by optogenetics stopped the pain behavior in mice (Mai et al., 2020). Due to its high accuracy and low side effects, optogenetics may be widely used in the field of pain in the near future.

#### Vision

Special neurons in the retina react to light and transmit it to the brain to produce vision (Pozarickij et al., 2020; Creeden et al., 2021; Liu H. et al., 2021; Yu et al., 2021; Elkhalifa et al., 2022). When neurons in the retina stop working properly, the eye can’t work properly. Nowadays, optogenetic can be used to treat eye diseases such as color blindness (Table 1 and Figure 3; Cideciyan et al., 2016). The researchers sensitized the cells to light by the expression of light genes that encode light-activated channels or pumps in the remaining retinal cells (Ostrovsky and Kirpichnikov, 2019; Blomeier et al., 2021; He et al., 2021; Kramer et al., 2021; Mickoleit et al., 2021). Relevant research has achieved good results in recent years. In 2017, Russian scientists injected drugs with certain genetic structure into the blind eyes of experimental rodents, and the sight of experimental animals was partially restored (Aung et al., 2017). Berry et al. (2019) restored vision to blind mice (Table 1 and Figure 3). In 2021, the company of Bionic Sight successfully used optogenetics to enable patients with advanced retinitis pigmentosa to see light and motion (Harris and Gilbert, 2022).

## Conclusion, Challenges and Perspectives

Since the advent of optogenetics, the technology has occupied the research field of neurobiology. Using optogenetics, researchers have decoded many neural circuits that cannot be decoded with other techniques. Such as social behavioral neural circuits, body temperature regulating neural circuits, spatial learning and memory circuits and so on. In view of the minimally invasive and high accuracy of optogenetics, optogenetics has a broad prospect in clinical treatment. Many irreversible diseases, especially neurodegenerative changes, can be solved by optogenetics. And the related research has entered the clinical trial stage.

Although optogenetics has achieved a lot in many fields, it still faces many challenges. Many scientists have proposed that exogenous light exposure causes neurons to respond in a non-physiological way, leading to incorrect physiological conclusions (Oh et al., 2021). And whether exogenous photosensitive proteins can have potential effects on nerve cells. Due to economic and other factors, optogenetics is mostly used in mouse experiments. Clinical trials of optogenetics are rare. Therefore, it will take a long time to prove that optogenetics can be widely applied to humans.

At present, optogenetics continues to flourish. Photogenetic treatments for retinal degeneration (Cehajic-Kapetanovic et al., 2019; Gilhooley et al., 2021) and pain (Harriott et al., 2021) are also in clinical trials. Researchers are also developing more precise (Adesnik and Abdeladim, 2021) and less invasive optogenetic devices, such as SOUL (Gong et al., 2020). At the same time, optogenetics has strong compatibility, it can be used to study a variety of diseases, such as diabetes (Li et al., 2021), inflammation (Baumschlager and Khammash, 2021; Bhat et al., 2021; Dos Santos et al., 2021; Jamaluddin et al., 2021; Michoud et al., 2021; Santos et al., 2021; Senok et al., 2021), tumors (Kim et al., 2017; Adampourezare et al., 2021; Esmaeili et al., 2022), depression (Hare et al., 2019), epilepsy (Zhang and Wang, 2021) and so on. And in 2020, 45 laboratories around the world integrated all optogenetics resources and create an optogenetics experimental database (Tremblay et al., 2020). According to the current progress, optogenetics has a broad prospect. It is believed that soon, optogenetics will become a major technique in neurobiology.

## Author Contributions

XZ and ZL designed this study and supervised the research. WC wrote the manuscript. WL, YLi, ZZ, YX, YLiao, LY, QL, and MH discussed the manuscript. CL, ZL, and XZ edited the manuscript. All authors read and approved the final manuscript.

## Conflict of Interest

The authors declare that the research was conducted in the absence of any commercial or financial relationships that could be construed as a potential conflict of interest.

## Publisher’s Note

All claims expressed in this article are solely those of the authors and do not necessarily represent those of their affiliated organizations, or those of the publisher, the editors and the reviewers. Any product that may be evaluated in this article, or claim that may be made by its manufacturer, is not guaranteed or endorsed by the publisher.
